# SNHG15 affects the growth of glioma microvascular endothelial cells by negatively regulating miR-153

**DOI:** 10.3892/or.2022.8267

**Published:** 2022-01-18

**Authors:** Yawen Ma, Yixue Xue, Xiaobai Liu, Chengbin Qu, Heng Cai, Ping Wang, Zhiqing Li, Zhen Li, Yunhui Liu

Oncol Rep 38: 3265-3277, 2017; DOI: 10.3892/or.2017.5985

Following the publication of this article, the authors have realized that they had inadvertently used the same western blotting data to show the GAPDH control western blots in Figs. 1C and 4D. In examining their original data, the authors realized that the data for Fig. 1C had been placed incorrectly in the figure.

The corrected version of Fig. 1, showing the correct GAPDH bands, is shown below. The authors sincerely apologize for the error made during the preparation of this Figure, thank the Editor for granting them the opportunity to publish this Corrigendum, and regret any inconvenience that this mistake may have caused.

## Figures and Tables

**Figure 1.**
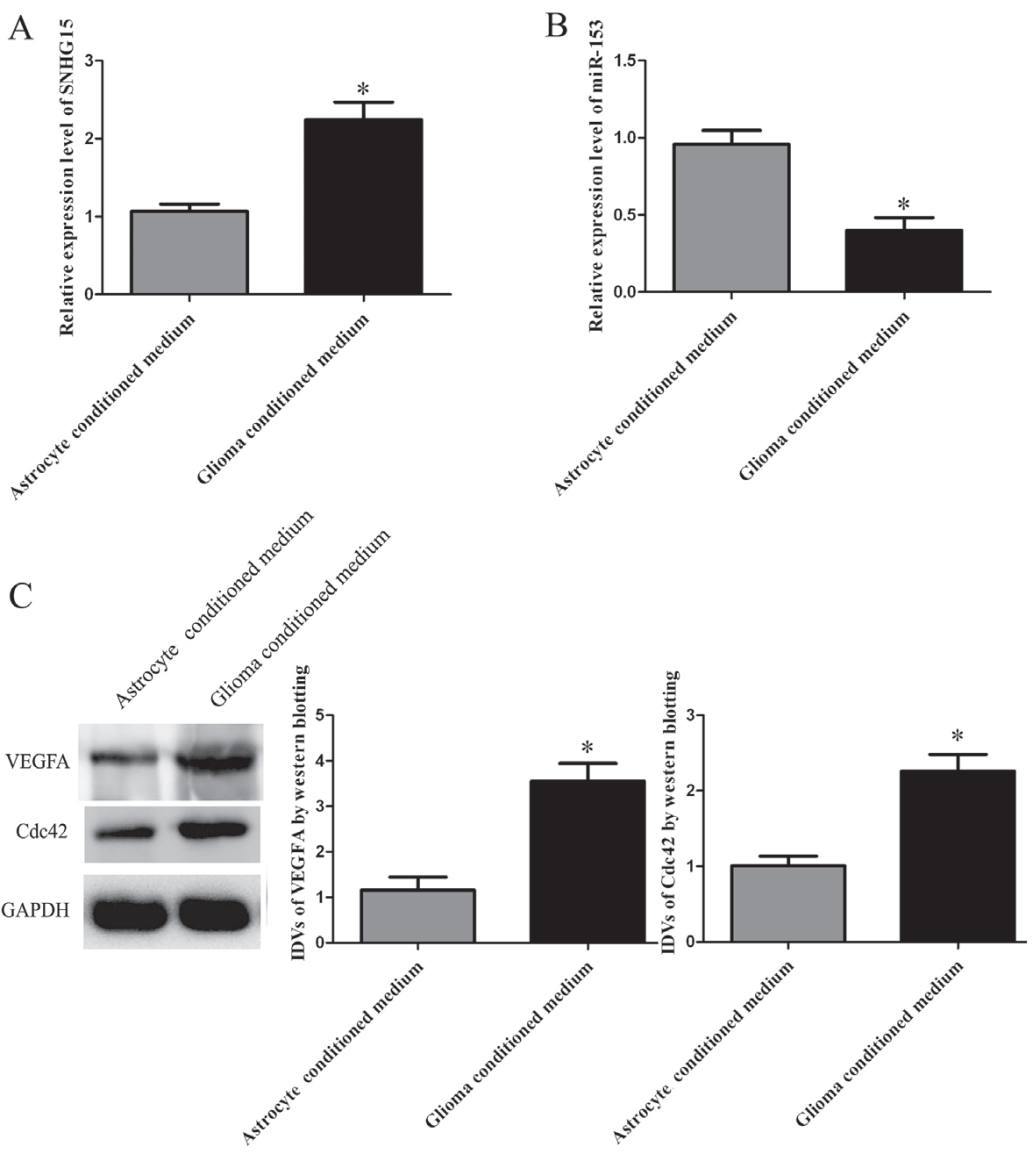
SNHG15, miR-153, VEGFA and Cdc42 expression in human cerebral microvascular endothelial cells (hCMECs) cultured in primary astrocyte cell conditioned and glioma conditioned medium. (A) Expression levels of SNHG15 in hCMECs cultured in primary astrocyte cell conditioned and glioma conditioned medium. (B) Expression levels of miR-153 in ECs cultured in primary astrocyte cell conditioned and glioma conditioned medium. (C) Western blot analysis of VEGFA and Cdc42 expression in hCMECs in primary astrocyte cell conditioned and glioma conditioned medium. The IDVs of VEGFA and Cdc42 are shown using GAPDH as an endogenous control. Data are presented as the mean ± SD (n=5, each group); ^*^P<0.05 vs. primary astrocyte cell conditioned medium group.

